# The Impact of the COVID-19 Pandemic on Time to Treatment in Surgical Oncology: A National Registry Study in The Netherlands

**DOI:** 10.3390/cancers16091738

**Published:** 2024-04-29

**Authors:** Roos M. G. van Vuren, Yester F. Janssen, Rianne N. M. Hogenbirk, Michelle R. de Graaff, Rinske van den Hoek, Schelto Kruijff, David J. Heineman, Willemijn Y. van der Plas, Michel W. J. M. Wouters

**Affiliations:** 1 Department of Surgery, University Medical Centre Groningen, 9713 GZ Groningen, The Netherlands; r.m.g.van.vuren@umcg.nl (R.M.G.v.V.); rianne.hogenbirk@mcl.nl (R.N.M.H.); m.r.de.graaff@umcg.nl (M.R.d.G.); r.van.den.hoek03@umcg.nl (R.v.d.H.); s.kruijff@umcg.nl (S.K.); 2 Department of Neurosurgery, University Medical Centre Groningen, 9713 GZ Groningen, The Netherlands; y.f.janssen@umcg.nl; 3 TRACER Europe B.V., Aarhusweg 2-1, 9723 JJ Groningen, The Netherlands; 4 Dutch Institute for Clinical Auditing, Scientific Bureau, 2333 AA Leiden, The Netherlands; 5 Department of Nuclear Medicine and Molecular Imaging, University Medical Centre Groningen, 9713 GZ Groningen, The Netherlands; 6 Department of Molecular Medicine and Surgery, Karolinska Institutet, 17177 Stockholm, Sweden; 7 Department of Surgery, Amsterdam UMC Location Vrije Universiteit Amsterdam, 1081 HV Amsterdam, The Netherlands; d.heineman@amsterdamumc.nl (D.J.H.); w.y.vanderplas@amsterdamumc.nl (W.Y.v.d.P.); 8 Department of Biomedical Data Sciences, Leiden University Medical Centre, 2333 ZA Leiden, The Netherlands; 9 Department of Surgical Oncology, Netherlands Cancer Institute-Antoni van Leeuwenhoek, 1066 CX Amsterdam, The Netherlands

**Keywords:** oncological surgery, COVID-19 pandemic, time to treatment

## Abstract

**Simple Summary:**

In The Netherlands, it has been agreed to start the treatment of cancer patients within six weeks of their first visit to the outpatient clinic. But in 2020, the care for COVID-19 patients and staff shortages have led to less capacity for regular surgical care. In this study, we used a national registry to investigate the impact of the COVID-19 pandemic on the time to start of treatment for cancer patients undergoing surgery. We found that, despite pressure on the healthcare system, more surgical cancer patients started treatment within six weeks, compared to the years before the COVID-19 pandemic. This could be because cancer care was given priority over other types of surgery. However, during the pandemic, fewer patients underwent surgery for cancer then before the pandemic. These were particularly patients with early-stage tumours. They might have received other treatments or were diagnosed with cancer later on.

**Abstract:**

To avoid delay in oncological treatment, a 6-weeks norm for time to treatment has been agreed on in The Netherlands. However, the impact of the COVID-19 pandemic on health systems resulted in reduced capacity for regular surgical care. In this study, we investigated the impact of the COVID-19 pandemic on time to treatment in surgical oncology in The Netherlands. Methods: A population-based analysis of data derived from five surgical audits, including patients who underwent surgery for lung cancer, colorectal cancer, upper gastro-intestinal, and hepato-pancreato-biliary (HPB) malignancies, was performed. The COVID-19 cohort of 2020 was compared to the historic cohorts of 2018 and 2019. Primary endpoints were time to treatment initiation and the proportion of patients whose treatment started within 6 weeks. The secondary objective was to evaluate the differences in characteristics and tumour stage distribution between patients treated before and during the COVID-19 pandemic. Results: A total of 14,567 surgical cancer patients were included in this study, of these 3292 treatments were started during the COVID-19 pandemic. The median time to treatment decreased during the pandemic (26 vs. 27 days, *p* < 0.001) and the proportion of patients whose treatment started within 6 weeks increased (76% vs. 73%, *p* < 0.001). In a multivariate logistic regression analysis, adjusting for patient characteristics, no significant difference in post-operative outcomes between patients who started treatment before or after 6 weeks was found. Overall, the number of procedures performed per week decreased by 8.1% during the pandemic. This reduction was most profound for patients with stage I lung carcinoma and colorectal carcinoma. There were fewer patients with pulmonary comorbidities in the pandemic cohort (11% vs. 13%, *p* = 0.003). Conclusions: Despite pressure on the capacity of the healthcare system during the COVID-19 pandemic, a larger proportion of surgical oncological patients started treatment within six weeks, possibly due to prioritisation of cancer care and reductions in elective procedures. However, during the pandemic, a decrease in the number of surgical oncological procedures performed in The Netherlands was observed, especially for patients with stage I disease.

## 1. Introduction

The COVID-19 pandemic had a major impact on health systems worldwide. A shift of focus of available resources towards COVID-19 care, in combination with staff shortages, compromised the capacity of regular surgical care. Many hospitals in The Netherlands adopted crisis strategies to optimize the use of limited resources [1]. Major surgical procedures necessitating post-operative ICU admission were delayed, due to the increased influx of COVID-19-related ICU admissions. In addition, reallocation of surgical nurses and doctors to COVID-19 wards reduced the capacity of regular wards and operating room capacity.

Postponing surgical cancer treatment may not be without consequences and may potentially lead to disease progression. Hence, during the first weeks of the pandemic, healthcare professionals expressed concerns about the potentially decreased survival rates among cancer patients due to the re-allocation of health resources to COVID-19 patients [2]. Additionally, a delay in cancer diagnosis was anticipated as screening programmes were temporarily halted and patients appeared to be reluctant to seek medical attention for early symptoms during that period. This might have been due to fear of contracting a COVID-19 infection in the hospital, or a wish to avoid adding pressure to the already overloaded healthcare system. A reduction in cancer diagnosis during the pandemic may have led to more advanced tumour stages later on, which could have led to worse oncological outcomes.

The impact of the COVID-19 pandemic on surgical care in The Netherlands has previously been investigated by de Graaff et al., who reported a 13.6% reduction in surgical procedures in the COVID-19 cohort of 2020 compared to the historical cohorts of 2018–2019 [3]. Patients who did receive surgical care during the pandemic had shorter waiting times. A decrease in time from diagnosis to surgery was observed for a wide range of surgical procedures (to an average of 28 days in 2020, from 34 days in 2019, and 36 days in 2018; *p* < 0.001) [3]. Time to treatment has not yet been investigated for oncological surgery specifically.

In The Netherlands, norms and standards have been agreed upon by the Dutch Federation of Oncological Societies (SONCOS) to minimize the delay in cancer care [4]. The recommendation in these standards is that patients should start treatment, e.g., surgical care or neo-adjuvant treatment, within six weeks after their first visit to the outpatient clinic. The aim of this study is to investigate if the recommended time to first treatment was met for lung, colorectal, upper GI, and HPB types of cancer during the COVID-19 pandemic in The Netherlands and—if not—what the consequences were on postoperative outcomes.

## 2. Materials and Methods

### 2.1. Study Design and Setting

This nationwide retrospective cohort study was performed with data of the national surgical registries at the Dutch Institute for Clinical Auditing: Dutch Colorectal Cancer Audit (DCRA), Dutch Upper-GI Cancer Audit (DUCA), Dutch Hepato Biliary Audit (DHBA), Dutch Pancreatic Cancer Audit (DPCA), and Dutch Lung Cancer Audit–Surgery (DLCA-S). These are mandatory, population-based, prospectively maintained quality registries that are accredited by national scientific boards and data quality is assured [5,6,7,8,9]. For this study, data from 50 hospitals were used (out of a total of 69 hospitals in The Netherlands [10]), that participated in the Dutch COVIDSurg II Snapshot Study [3]. According to Dutch law, no ethical approval was required as data was handled de-identified.

### 2.2. Study Population

**Figure 1:**
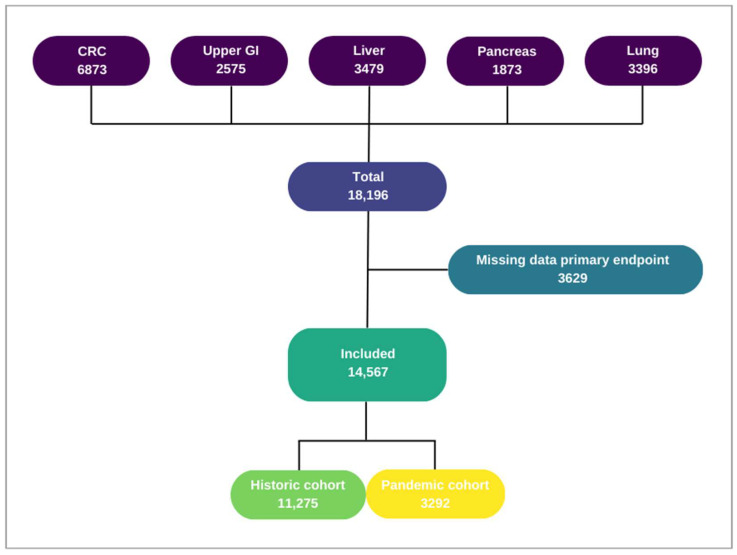
Flowchart included patients.

The respective registries include all patients with resection of primary colorectal carcinoma [11]; resection of primary or recurrent oesophageal or gastric carcinoma [12]; liver resection for hepatic metastasis of any origin, for primary liver malignancies (e.g., HCC), and for biliary cancer [13]; resection of pancreatic or periampullary cancers (or exploration with intention to resection) [14]; and resection of malignancies of the lung [15]. The combined database includes patients who underwent surgery between 1 January 2018 and 31 December 2020. Patients with missing data that hindered the calculation of primary endpoint ‘time to treatment’ were excluded. These were 3629 patients with a proportionally equal distribution over the historic and pandemic cohorts. The inclusion process is presented in Figure 1. For the analysis of tumour stage distribution, 1571 patients with T0 or Tx tumours were excluded, as were 1908 procedures for hepatic metastasis, because a shift in distribution is not possible for this group that is stage IV by definition.

### 2.3. Outcomes

Primary endpoints were the time to treatment initiation and the proportion of patients whose time to treatment met the 6-week norm as recommended by the Dutch Federation of Oncological Societies (SONCOS) [4]. Time to treatment was calculated as the number of days between first visit and date of surgery, or, when neo-adjuvant treatment was given, the number of days between first visit and start of neo-adjuvant treatment. Patients who underwent emergency surgery were excluded from the analysis of the primary endpoints because their time to surgery could not be seen as a measure of meeting the standards. The secondary objective was to evaluate the effects of (not) meeting the 6-week norm on post-operative outcomes such as length of hospital stay, 30-day mortality, and complete resections. Furthermore, a secondary objective was to assess differences in patients’ characteristics between the historic and pandemic cohorts, including tumour stage distribution.

### 2.4. Variables

Variables registered in the databases include demographic data and comorbidities, treatment characteristics, treatment dates, and post-operative outcomes. The Charlson Comorbidity Index score was calculated based on registered comorbidities and age in the year of surgery [16]. Emergency surgery was defined as a time between first visit and surgery of 3 days or less, or being registered as acute surgery regardless of calculated time to surgery. Severe post-operative complications were classified as Clavien Dindo grade 3 or higher [17].

### 2.5. COVID-19 Waves

**Figure 2:**
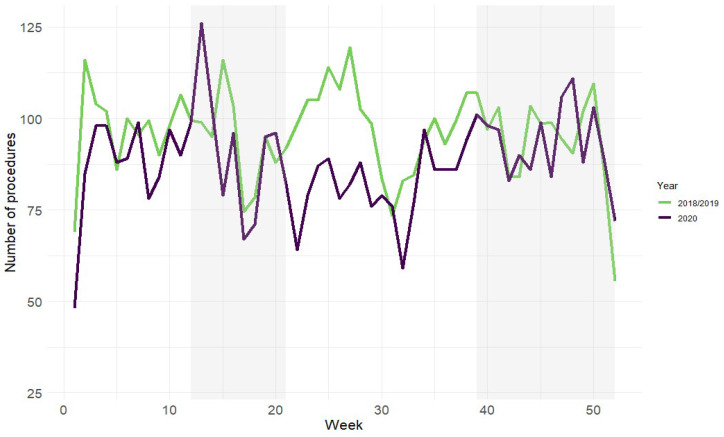
Number of procedures per week; pandemic waves depicted in grey.

In accordance with the timeline established by the Central Bureau for Statistics of The Netherlands [18], a delineation of four distinct periods was made based on COVID-19 hospital admission rates in The Netherlands. A particular day was classified within a ‘wave’ when at least 500 patients were hospitalized due to COVID-19, and/or a minimum of 200 COVID-19 patients were admitted to ICUs within The Netherlands [19]. The ‘first wave’ spanned from 16 March to 24 May, and the ‘second wave’ began on 21 September 2020, extending well into 2021 (Figure 2). To align with the study’s scope, the end of the second wave was set at 31st December 2020, as patients undergoing surgery in 2021 were not included. The period preceding these waves is referred to as ‘pre-pandemic’, while the timeframe in between is termed the ‘interim period’. For the analysis of time to treatment, patients were categorized into these groups based on the expected treatment initiation within the 6-week norm [4]. In specific tables, the ‘pandemic cohort’ encompasses the first wave, interim period, and second wave, aiming to present findings more comprehensively.

### 2.6. Statistical Analysis

Descriptive statistics were used to compare patient characteristics, treatment details and time to treatment between the historic and pandemic cohorts. Categorical and binary variables are presented as numbers with percentages and were compared using Pearson’s Chi-squared test. Numerical variables are presented as median and interquartile range, and were compared using the Mann–Whitney U test or Kruskal–Wallis rank sum test for multiple groups. Logistic modelling was used to calculate the odds ratio with a 95% confidence interval for the likelihood of meeting the 6-week norm; adjusted for tumour type and stage and Charlson Comorbidity Index. No imputation was performed. Data were analysed using RStudio version 4.2.3 (R Foundation for Statistical Computing, Vienna, Austria, 2023). The package gtsummary was used for creating tables and ggplot2 for creating graphs.

## 3. Results

### 3.1. Patient and Treatment Characteristics

A total of 14,567 surgical cancer patients were included in this study, of these 3292 treatments were started during the COVID-19 pandemic. A decrease in number of procedures of 8.1% was observed in 2020 as compared to the historical cohorts of 2018/2019, see Figure 2 for a timeline of the weekly number of procedures to a background of the first and second wave of the pandemic. No statistically significant differences were found in age, gender, and BMI between the two cohorts (Table 1). During the COVID-19 pandemic, fewer patients who underwent surgery had pulmonary comorbidities (11% vs. 13%, *p* = 0.003). A difference was observed in the Charlson Comorbidity Index (CCI) and ASA classification: patients in the pandemic cohort had a higher CCI (4.5 vs. 4.1, *p* < 0.001) and were more often classified as ASA 3 (36% vs. 33%, *p* = 0.006) and less often as ASA 1 or 2 (62.4% vs. 65.5%, *p* = 0.006). Further characteristics of patients are described in Table 1.

Table 2 shows the treatment characteristics during different periods of the pandemic. An increase in the proportion of emergency surgery was seen, especially during the first pandemic wave (15% vs. 10% in the pre-pandemic period, *p* < 0.001). Furthermore, during the first wave, more procedures were performed open and fewer laparoscopically, compared to the historic cohort (open 31% vs. 27%, laparoscopically 53% vs. 57%, *p* = 0.016). The most profound decrease in the number of procedures performed per week was observed in lung cancer surgery, while for pancreatic cancer patients an increase in number of procedures was seen during the first wave of the pandemic (weekly number 7.3 vs. 5.3, *p* = 0.032).

### 3.2. Time to Treatment

The median time between the first visit and the start of treatment decreased after the first wave of the COVID-19 pandemic. Time to treatment was shortest during the interim period with 25 days as compared to 27 days before the pandemic (*p* < 0.001). Adjusted for tumour type, tumour stage, and Charlson Comorbidity Index, patients were more likely to start treatment within 6 weeks of their first visit during the interim period and second wave compared to the pre-pandemic period (second wave OR 1.50, CI 1.22–1.86, *p* < 0.001). In Table 3, the time to treatment and the percentage of patients starting treatment within 6 weeks are detailed for different tumour groups.

### 3.3. Postoperative Outcomes

**Figure 3:**
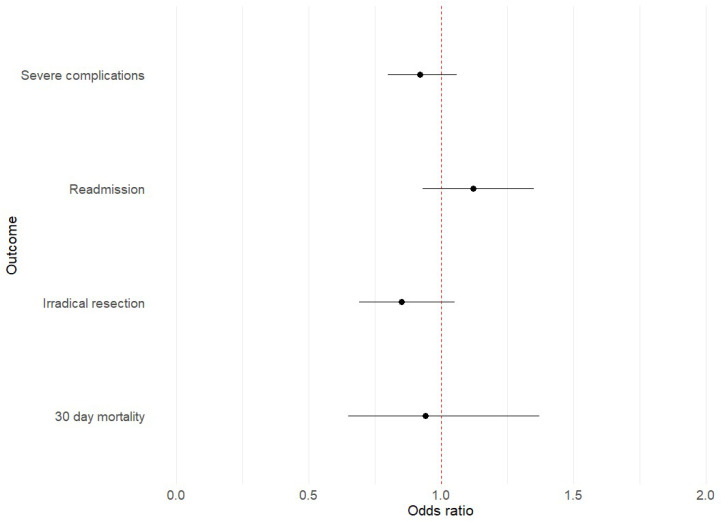
Forest plot of adjusted odds ratio and confidence interval of post-operative outcomes in patients with delayed treatment (>6 weeks); adjusted for age, sex, tumour type, tumour stage, ASA classification, and pulmonary comorbidity.

When comparing the postoperative outcomes of patients who did and did not start treatment within the 6-weeks norm, there was no difference in length of hospital stay and readmission within 30 days. There was a difference in mortality rate and complete resections between the groups, but when corrected for age, sex, tumour type and stage, ASA classification, and pulmonary comorbidity, the effect was not significant. The adjusted odds ratio for 30-day mortality was 0.94 (CI 0.65–1.37, *p* 0.7) for the >6 w group as compared to the <6 w group. The adjusted odds ratio for an incomplete resection (R1 or R2) was 0.85 (CI 0.69–1.05, *p* 0.12) for the >6 w group as compared to the <6 w group. See Table 4 and Figure 3.

### 3.4. Tumour Stage Distribution

**Figure 4:**
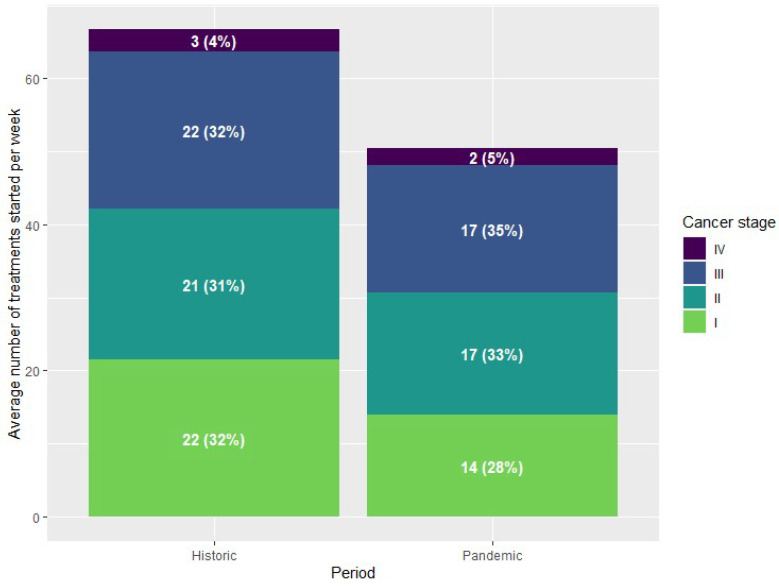
Stage distribution and tumour types of surgical patients.

**Figure 5:**
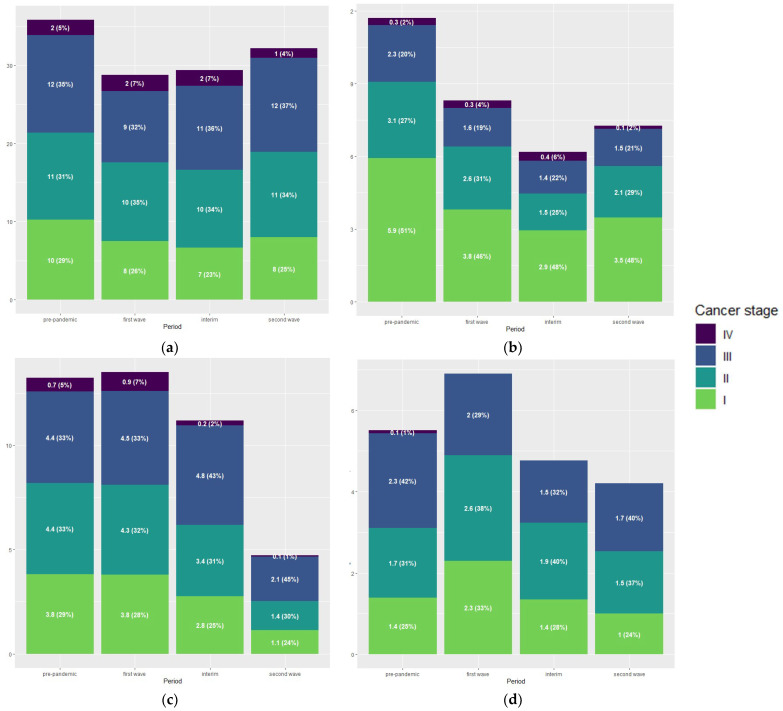
Stage distribution by tumour type and period of pandemic of surgical patients: (**a**) colorectal carcinoma; (**b**) lung carcinoma; (**c**) gastric and oesophageal carcinoma; (**d**) pancreatic carcinoma.

Of all tumour stages, the highest decrease was observed in the weekly number of performed procedures for patients with stage I tumours, with 14 procedures per week during the pandemic compared to 22 per week in the historic cohort. This effect was most profound in procedures for lung carcinoma (3 vs. 6 procedures per week, *p* < 0.001) and colorectal carcinoma (7 vs. 10 procedures per week, *p* = 0.014). Figure 4 shows the distribution of cancer stages for all tumour types combined, while Figure 5 details the stage distribution per tumour type and period of the pandemic. The weekly number of procedures for stage I and II pancreas tumours increased during the first wave, while the number of operated stage III tumours decreased. For upper GI surgery no change in stage distribution was observed and even though the data shows a steep reduction of treatments started during the second wave this is not a true observation but a consequence of the structure of the dataset. There was no evidence of an increase in advanced tumour stages in any of the tumour types.

## 4. Discussion

In The Netherlands, the time to treatment in surgical oncology decreased significantly during the COVID-19 pandemic compared to the historical period of 2018–2019. A higher proportion of patients started treatment within six weeks of their initial outpatient visit, as recommended by the Dutch Federation of Oncological Societies. In this cohort, patients starting treatment later than six weeks had no significant disadvantage in terms of incomplete resections or 30-day mortality. The overall number of procedures decreased during the COVID-19 pandemic, most significantly for stage I tumours, particularly among lung- and colorectal cancer patients. Compared to the historical cohort of 2018–2019, patients receiving oncological surgery during the pandemic less often had pulmonary comorbidities and required emergency surgery more frequently.

This is the first population-based study evaluating time to treatment initiation during the COVID-19 pandemic for multiple tumour types in a European setting. In Canada, Fu et al. conducted a population-based study including multiple tumour types, which also found a reduction in the mean waiting time for surgery, chemotherapy, and radiotherapy [20]. The finding in our study that time to treatment initiation decreased during the pandemic was confirmed by several population-based studies focusing on other tumour types [21,22,23,24,25]. Notably, the largest reduction was seen in bladder cancer care with a reduction of 6 days from diagnosis to radical cystectomy [23]. In contrast, two population-based HPB studies in The Netherlands and the United States found no reduction in time to treatment initiation [26,27]. In our cohort, the faster time to start of treatment for oncological patients during the pandemic is probably the result of the prioritization of oncological care in The Netherlands. Despite the pressure on the healthcare system, the postponement of elective non-oncological procedures (e.g., bariatric, hernia, or joint replacement surgery) might have created treatment capacity for oncological care. In non-surgical cancer care in The Netherlands, a similar effect of decreased time to treatment initiation was observed in radiotherapy for head and neck cancers [28], hormonal treatment for breast cancer [29], and neo-adjuvant treatment for colorectal cancer [22].

As a secondary endpoint, we investigated the impact of delayed treatment initiation, defined by the Dutch Federation of Oncological Societies [4] as not commencing treatment within six weeks from diagnosis, on short term post-operative outcomes. Our initial hypothesis was that prolonged time to treatment could potentially heighten the risk of disease progression, thereby either leading to more frequent positive resection margins or prompting more extensive resections associated with increased complication risks. However, after correcting for confounding factors such as comorbidities and tumour stage, our analysis revealed that time to treatment initiation exceeding 6 weeks had no significant effect on the incidence of severe complications, readmission, irradical resections, or 30-day mortality. However, it was not possible to study the effect on long term outcomes such as survival in this dataset. The six-week timeframe recommended by the Dutch Federation of Oncological Societies lacks a clear scientific rationale. Three recent systematic reviews have summarised the evidence of the effect of time to surgery on survival in gastrointestinal and thoracic cancers [30,31,32]. While there is evidence that increased time to surgery adversely impacts survival in some tumour types, the studied timeframes differ substantially and provide no basis for a 6-weeks norm for all tumour types and stages. This gives room for debate on the importance of abiding to rigid time to treatment norms in times of healthcare scarcity, taking into account that in certain cases patients can benefit from prehabilitation [33]. Nonetheless, it can be difficult for patients to have to cope with waiting times after receiving a cancer diagnosis.

Our results show a reduction in surgical procedures for stage I tumours, especially in lung cancer and colorectal cancer patients, which is in line with the findings of other population-based studies [34,35]. This suggests a subset of individuals who might have received surgery under normal circumstances, but faced altered treatment decisions or remained undiagnosed during the pandemic. The decrease in early stage colorectal tumours is likely due to the temporary cessation of the national colorectal cancer screening programme, causing a decrease in the detection of asymptomatic stage I tumours [34,36]. However, this effect was not found in lung cancer procedures in Japan, where Sato et al. reported no difference in tumour staging despite interruption of the lung cancer screening programme [37]. We hypothesize that the reduction in procedures for stage I lung tumours in our cohort was caused by a shift in treatment strategies from surgery to stereotactic radiation, since there is no screening programme for lung cancer in The Netherlands. This will be investigated in a future study in this specific cohort.

We also observed a rise in emergency surgical procedures, particularly evident during the first wave of the pandemic, where 15% of procedures were conducted urgently, predominantly among colorectal cancer (CRC) patients. Previous population-based studies including CRC patients reported similar findings or found no difference in emergency procedures [35,38]. The increase in emergency procedures among CRC patients might be attributed to a shift towards more advanced disease at the time of diagnosis, when a reduced number of patients were diagnosed with early-stage tumours during the pandemic. Our dataset has no information about the symptoms of patients requiring emergency surgery. However, Meijer et al. conducted a study utilizing the Dutch CRC diagnoses database that partly overlaps with our dataset, and revealed a significant increase in clinical presentations with ileus towards the end of the first pandemic wave compared to the beginning of 2020 (14.3% vs. 8%, *p* < 0.01) [22]. This observation may potentially clarify our findings.

The main strength of this study is that it uses nationwide data from an obligatory surgical audit, thereby accurately reflecting daily practice. This study not only compared median time to treatment before and during the pandemic, but also performed logistic regression to assess the probability of treatment initiation within six weeks. This allows correction for a changed patient population during the pandemic and observation of the true impact of changes in organization of care. However, this study also has certain limitations. The dataset combined data from several audits, but not all variables of the original data were available in this dataset, for example, the presence of ileus in CRC patients and long-term survival data. Also, for lung cancer patients, the time to surgery was calculated using the first visit with their pulmonologist instead of first visit with the oncologic surgeon like the other audits did. This partially explains the longer time to surgery of lung cancer patients compared to other tumour types; however, it does not introduce bias because the method of calculation was the same in the historic and pandemic cohort. Another limitation lies in the fact that 20% of patients of the initial dataset were excluded from analysis in this study because of missing data on the primary endpoint. Yet, it is important to note that the distribution over the historic and pandemic cohort was proportionally equal; this implies minimal potential for influencing our findings. Moreover, there is a limitation in the categorization of patients in different time periods of the pandemic. The dataset’s inclusion criteria were based on the surgery date falling between 2018 and 2020. Consequently, patients who started neo-adjuvant treatment in 2020, but underwent surgery in 2021 are not incorporated. Especially for upper GI tumours, this results in an inaccurate depiction of the number of treatments started during the second wave. Finally, the inclusion criteria were limited to patients undergoing surgery, so conclusions cannot be drawn regarding oncological patients with alternative treatment plans. It is important to note that our findings on tumour stage distribution do not include all diagnosed patients but only surgical patients. Therefore, results should be interpreted with care.

## 5. Conclusions

Our study revealed that—despite the strain on healthcare system capacity during the pandemic—a higher proportion of patients started treatment within 6 weeks from their initial outpatient clinic visit. Dutch hospitals, facing challenges posed by the COVID-19 pandemic, responded by prioritizing oncological surgeries above treatment of benign conditions. The deferral of elective procedures likely facilitated the availability of diagnostic and treatment resources for oncological care. While this prioritization positively impacted oncological patients, further research is essential to contrast its effects on those awaiting elective surgeries. Additionally, our study indicated a decline in surgical oncological procedures, particularly among patients with stage I disease. Some of these patients might have received alternative treatments, such as radiation therapy. Exploring the long-term effects of these altered treatment strategies would be valuable. Learning lessons from our adaptation strategies during the pandemic period will ultimately aid in improving the strength and resilience of our healthcare system that will face many challenges to come.

## Figures and Tables

**Table 1:** Patient characteristics.

Characteristic	Historic Cohort, N = 11,275	Pandemic Cohort, N = 3292	*p*-Value ^1^
Age (years) Missing	68 (10.6)	68 (10.8)	0.9 ^§^
	<0.1%	0.2%	
Female sex Missing	4712 (42%)	1403 (43%)	0.4
	-	<0.1%	
BMI > 30 Missing	2024 (19%)	577 (18%)	0.3
	4%	2%	
Pulmonary comorbidity Missing	1360 (13%)	373 (11%)	0.003
	10%	0.5%	
Charlson Comorbidity Index	4.1 (2.3)	4.5 (2.5)	<0.001 ^§^
Missing	<0.1%	0.2%	
ASA classification			0.006
1	1064 (9.4%)	273 (8.3%)	
2	6325 (56.4%)	1774 (54.5%)	
3	3651 (32.5%)	1160 (35.7%)	
4	188 (1.7%)	49 (1.5%)	
Missing	0.4%	1%	
Tumour type			<0.001
CRC	4369 (38.8%)	1282 (39%)	
HPB	3357 (29.8%)	1169 (35.5%)	
Lung	1556 (13.8%)	347 (10.5%)	
Upper GI	1993 (17.6%)	494 (15%)	
Cancer stage ^2^			<0.001
I	2459 (32.3%)	587 (27.8%)	
II	2343 (30.8%)	703 (33%)	
III	2467 (32.4%)	734 (34.6%)	
IV	339 (4.5%)	98 (4.6%)	
Missing	11%	9%	

Data are presented as mean (SD) and number (%), ^1^ Pearson’s Chi-squared test, except ^§^ Mann–Whitney U test, ^2^ Excluding procedures for hepatic metastasis, T0 and Tx.

**Table 2:** Treatment characteristics.

Characteristic	Pre-Pandemic, N = 10,935	First Wave, N = 920	Interim, N = 1377	Second Wave, N = 1335	*p*-Value ^1^
Emergency surgery ^2^ Missing	619 (10%)	77 (15%)	99 (13%)	67 (8.9%)	<0.001
	43%	44%	44%	44%	
Neoadjuvant treatment Missing	1581 (16%)	195 (23%)	250 (20%)	224 (18%)	<0.001
	8.0%	7.4%	8.9%	8.4%	
Surgical approach Open					0.2
	2850 (27%)	275 (30.7%)	374 (28%)	331 (25.4%)	
Laparoscopic Conversion	5965 (56.6%)	474 (52.7%)	726 (54.6%)	752 (58%)	
	727 (6.9%)	58 (6.5%)	93 (7.0%)	80 (6.2%)	
Robot assisted	308 (2.9%)	29 (3.2%)	52 (3.9%)	46 (3.5%)	
Local/ablation	691 (6.6%)	62 (6.9%)	86 (6.5%)	90 (6.9%)	
Missing	3.6%	2.4%	3.3%	2.7%	

Data are presented as number (%), Historic cohort: 1 January 2018 to 14 March 2020; Pandemic cohort: 15 March 2020 to 31 December 2020; based on date of surgery, ^1^ Pearson’s Chi-squared test, ^2^ Time between first visit and surgery of 3 days or less, or registered as acute surgery.

**Table 3:** Time to treatment.

Characteristic	Pre-Pandemic, N = 10,480	First Wave, N = 789	Interim, N = 1222	Second Wave, N = 963	*p*-Value
Time to treatment (days)	27 (16, 43)	27 (15, 43)	25 (14, 39)	26 (15, 39)	<0.001 ^1^
CRC	19 (12, 29)	15 (9, 31)	16 (10, 28)	18 (11, 28)	0.01
HPB	33 (21, 50)	31 (20, 47)	29 (19, 46)	30 (20, 42)	<0.001
Upper GI	22 (15, 34)	26 (15, 36)	21 (15, 33)	26 (17, 40)	0.2
Lung	50 (36, 69)	48 (34, 63)	46 (33, 63)	42 (31, 60)	<0.001
Treatment started < 6 w	7636 (73%)	573 (73%)	945 (77%)	757 (79%)	<0.001 ^2^
CRC (N = 4942)	90%	84%	91%	92%	0.01
HPB (N = 4387)	66%	68%	71%	73%	0.009
Upper GI (N = 2226)	83%	83%	88%	77%	0.1
Lung (N = 1899)	35%	41%	42%	47%	0.02

Data are presented as median (IQR) and number (%), Historic cohort: 1 January 2018 to 14 March 2020; Pandemic cohort: 15 March 2020 to 31 December 2020; patients were categorized into these groups based on the expected treatment initiation within the 6-week norm. Excluding emergency surgery, ^1^ Kruskal–Wallis rank sum test; ^2^ Pearson’s Chi-squared test.

**Table 4:** Postoperative outcomes of patients who did and did not start treatment within 6 weeks.

Cohort	Historic			Pandemic		
Outcome	<6 w, N = 7734	>6 w, N = 2920	*p*-Value ^1^	<6 w, N = 2322	>6 w, N = 729	*p*-Value ^1^
Length of hospital stay (days)	7 (4, 11)	7 (4, 11)	0.7	6 (4, 10)	7 (4, 10)	0.4
Missing	0.4%	0.6%		0.8%	1.0%	
Readmission within 30 days	745 (10%)	254 (9.6%)	0.2	220 (9.6%)	75 (11%)	0.5
Missing	8%	10%		2%	2%	
Severe complication	1253 (16%)	492 (17%)	0.4	360 (16%)	123 (17%)	0.4
Missing	0.3%	0.7%		0.1%	0	
30-day mortality	124 (1.6%)	77 (2.6%)	<0.001	48 (2.1%)	13 (1.8%)	0.6
Missing	0.4%	0.4%		1.0%	1.0%	
Resection margins			0.003			0.04
R0	6065 (93.7%)	2000 (92.1%)		1732 (91.5%)	459 (88.3%)	
R1	397 (6.1%)	159 (7.3%)		150 (7.9%)	59 (11.3%)	
R2	16 (0.2%)	14 (0.6%)		12 (0.6%)	2 (0.4%)	
Missing	16%	26%		18%	29%	

Data are presented as median (IQR) and number (%), ^1^ Wilcoxon rank sum test; Pearson’s Chi-squared test.

## Data Availability

The datasets used and/or analysed during the current study are available from the corresponding author on reasonable request.
